# Rapid and simple colorimetric detection of multiple influenza viruses infecting humans using a reverse transcriptional loop-mediated isothermal amplification (RT-LAMP) diagnostic platform

**DOI:** 10.1186/s12879-019-4277-8

**Published:** 2019-08-01

**Authors:** Su Jeong Ahn, Yun Hee Baek, Khristine Kaith S. Lloren, Won-Suk Choi, Ju Hwan Jeong, Khristine Joy C. Antigua, Hyeok-il Kwon, Su-Jin Park, Eun-Ha Kim, Young-il Kim, Young-Jae Si, Seung Bok Hong, Kyeong Seob Shin, Sungkun Chun, Young Ki Choi, Min-Suk Song

**Affiliations:** 1 Department of Microbiology, College of Medicine and Medical Research Institute, Chungbuk National University, Chungdae-ro 1, Seowon-Ku, Cheongju, 28644 Republic of Korea; 2 Department of Clinical Laboratory Science, Chungbuk Health and Science University, Cheongju, Republic of Korea; 3 Departments of Laboratory Medicine, Chungbuk National University College of Medicine, Cheongju, Republic of Korea; 4 Department of Physiology, Chonbuk National University Medical School, Jeonju, 54907 Republic of Korea

**Keywords:** RT-LAMP, Seasonal influenza, Avian influenza, Multiplex detection, Colorimetric visualization

## Abstract

**Background:**

In addition to seasonal influenza viruses recently circulating in humans, avian influenza viruses (AIVs) of H5N1, H5N6 and H7N9 subtypes have also emerged and demonstrated human infection abilities with high mortality rates. Although influenza viral infections are usually diagnosed using viral isolation and serological/molecular analyses, the cost, accessibility, and availability of these methods may limit their utility in various settings. The objective of this study was to develop and optimized a multiplex detection system for most influenza viruses currently infecting humans.

**Methods:**

We developed and optimized a multiplex detection system for most influenza viruses currently infecting humans including two type B (both Victoria lineages and Yamagata lineages), H1N1, H3N2, H5N1, H5N6, and H7N9 using Reverse Transcriptional Loop-mediated Isothermal Amplification (RT-LAMP) technology coupled with a one-pot colorimetric visualization system to facilitate direct determination of results without additional steps. We also evaluated this multiplex RT-LAMP for clinical use using a total of 135 clinical and spiked samples (91 influenza viruses and 44 other human infectious viruses).

**Results:**

We achieved rapid detection of seasonal influenza viruses (H1N1, H3N2, and Type B) and avian influenza viruses (H5N1, H5N6, H5N8 and H7N9) within an hour. The assay could detect influenza viruses with high sensitivity (i.e., from 100 to 0.1 viral genome copies), comparable to conventional RT-PCR-based approaches which would typically take several hours and require expensive equipment. This assay was capable of specifically detecting each influenza virus (Type B, H1N1, H3N2, H5N1, H5N6, H5N8 and H7N9) without cross-reactivity with other subtypes of AIVs or other human infectious viruses. Furthermore, 91 clinical and spiked samples confirmed by qRT-PCR were also detected by this multiplex RT-LAMP with 98.9% agreement. It was more sensitive than one-step RT-PCR approach (92.3%).

**Conclusions:**

Results of this study suggest that our multiplex RT-LAMP assay may provide a rapid, sensitive, cost-effective, and reliable diagnostic method for identifying recent influenza viruses infecting humans, especially in locations without access to large platforms or sophisticated equipment.

**Electronic supplementary material:**

The online version of this article (10.1186/s12879-019-4277-8) contains supplementary material, which is available to authorized users.

## Background

The World Health Organization constantly monitors global influenza activity. It has noted global circulation of H1N1, H3N2, and two lineages of type B (Victoria- and Yamagata-) viruses that cause 670,000 annual deaths worldwide [1]. In the past few years, human infections with several subtypes of avian influenza virus (AIV) (e.g., H5 and H7) occurred sporadically [2]. Highly pathogenic avian influenza (HPAI) H5N1 and H5N6 viruses have infected 878 humans with 53% mortality since 1997 [3]. Since the first case of human infection with low pathogenic avian influenza (LPAI) H7N9 virus reported in China in 2013, the number of humans infected with this virus has dramatically increased to more than 1,567 as of April 2019 [4]. Recognizing this public concern, it is important to distinguish seasonal influenza and avian influenza viruses such as H5 and H7N9 that occur simultaneously in humans, especially in China.

Vaccination and treatment with antiviral drugs (e.g., neuraminidase inhibitors (NAIs)) are primary interventions to prevent viral infections and their spread. However, vaccine production usually takes 6–12 months to prepare for newly emerging viruses. NAIs also should be taken within the first 48 h following an infection [5]. Thus, rapid and accurate diagnosis of viral infections is important for mitigating the spread of virus within a community, facilitating immediate treatment with NAIs, and controlling carriers of these pathogens. Methods to detect and identify influenza viruses have improved over the past decades, ranging from traditional virus culture [6] to introduction of serological and molecular diagnostic technology (e.g., real-time RT-PCR [7] and PCR [8]) and more recently, rapid influenza detection tests (RIDT) [9–11]. With various influenza-specific diagnostic tools, selecting the most appropriate approach is based on a number of factors, including sensitivity, specificity, throughput, cost, and availability [12].

Virus isolation and serology method has traditionally been used to detect influenza virus. However, it may take several days to obtain results [13]. RIDT is a rapid method of performing point-of-care testing (POCT) in the field. Most RIDT diagnostic kits can detect influenza nucleoprotein antigen [14]. However, RIDT has two potential limitations: i) relatively large numbers of influenza viruses must be present for accurate detection in the collected sample, and ii) inability to distinguish between influenza subtypes. Overall, sensitivity and accuracy of RIDT are lower than those of qRT-PCR-based methods that not only can amplify small amounts of target viral RNA, but also can allow for determination of influenza A virus subtype using specifically designed primers [12]. Despite qRT-PCR-based approaches have these advantages, they typically take at least a few hours up to 2 days to obtain results [15]. In addition, qRT-PCR needs trained personnel and sophisticated facilities for sample processing. These disadvantages limit its function in ensuring rapid prescription and administration of antiviral agents to patients. Recently, many Clinical Laboratory Improvement Amendments (CLIA)-waived molecular tests have been approved for point-of-care use (https://www.cdc.gov/flu/professionals/diagnosis/molecular-assays.htm). Although the detection time (similar to RIDT) and sensitivity (better than RIDT) of CLIA-waived molecular tests have been improved, they are mostly limited to seasonal flu detection. They are incapable of discriminating seasonal influenza of avian subtypes that can cause human infection [16].

Loop-mediated isothermal amplification (LAMP) assays can amplify specific nucleic acids at a consistent temperature. They have been used for rapid detection of specific genes [17–20]. In particular, LAMP method combined with reverse transcription (called RT-LAMP) is a method for simultaneously synthesizing cDNA from template RNA and amplifying DNA [21]. Thus, RT-LAMP is useful for detecting RNA viruses. In addition, polymerase enzyme produces protons and subsequently leads to decreased pH in the presence of extensive DNA polymerase activity during LAMP reaction, thus facilitating real-time and simple detection of amplicons as observed by a change from pink to yellow color in the reaction solution [22]. The specificity and sensitivity of RT-LAMP are potentially comparable to those of existing PCR-based diagnostics with much shorter reaction time. Recently, influenza diagnostics tools leveraging RT-LAMP technology have been reported [23–30]. However, most of these tools can only diagnose a single or a small number of human influenza viruses. Their ability to diagnose newly emerging viruses is limited. None of these methods can simultaneously differentiate infection of seasonal influenza from multiple avian influenza virus infection including recently emerging H5Nx and H7N9, although this is critical when infection by those viruses to humans simultaneously occurs.

Thus, the objective of this study was to develop a multiplex RT-LAMP diagnostic method capable of simultaneously detecting human influenza viruses (two lineages of type B, H1N1 and H3N2) currently circulating in humans and avian influenza viruses (H5N1, H5N6 and H7N9) in rapidly emerging human infections. This assay also involves a one-pot colorimetric visualization approach that allows for direct observation by the naked eye. Overall, this diagnostic assay may be useful as a rapid and highly sensitive POCT that requires no laboratory equipment for field-based applications.

## Methods

### Viruses and viral titration

A/California/04/2009 (H1N1pdm), A/Anhui/1/2013 (H7N9), A/Perth/16/2009 (H3N2), B/Brisbane/60/2008 (Victoria lineage), and B/Phuket/3073/2013 (Yamagata lineage) were used for evaluating the RT-LAMP assay. A/Em/Korea/W149/2006 (H5N1) and A/Em/Korea/w468/2014 (H5N8) viruses were isolated from wild bird feces in South Korea [31, 32]. Candidate vaccine strains containing Hemagglutinin (HA) and neuraminidase (NA) genes from A/Sichuan/26221/2014 (H5N6) and A/gyrfalcon/Washington/41088–6/2014 (H5N8) in A/Puerto Rico/8/34 were kindly provided by World Health Organization (WHO). Handling of highly pathogenic avian influenza H5 viruses and H7N9 virus were conducted in an enhanced biosafety level 3 (BSL-3+) facility approved by Korea Centers for Disease Control and Prevention (KCDC). MERS-CoV Korean isolate (MERS-CoV/KOR/KNIH/002 _05_2015, GenBank accession no. KT029139) was kindly provided by KCDC. To determine virus infectious titers, 50% tissue culture infectious dose (TCID_50_) assay was performed in MDCK cells. Each virus was individually prepared by serial 10-fold dilutions in infection medium (minimal essential medium). The prepared virus diluent was used to infect MDCK cells in 96-well plates and incubated at 37 °C for 1 h in a CO_2_ incubator. Supernatants were then replaced with infection medium (MEM) with 1 μg/L L-(tosylamido-2-phenyl) ethyl chloromethyl ketone (TPCK)-treated trypsin. After 2~3 days of incubation, virus titers were determined using the hemagglutination (HA) assay with 0.5% turkey or chicken red blood cells (RBCs). Then 200 μl of virus stock was used to extract viral RNA using RNeasy Mini Kit (Qiagen, Hilden, Germany) according to the manufacturer’s protocol. After extraction, RNA was stored at − 80 °C until use.

### RT-LAMP primer design

**Fig. 1:**
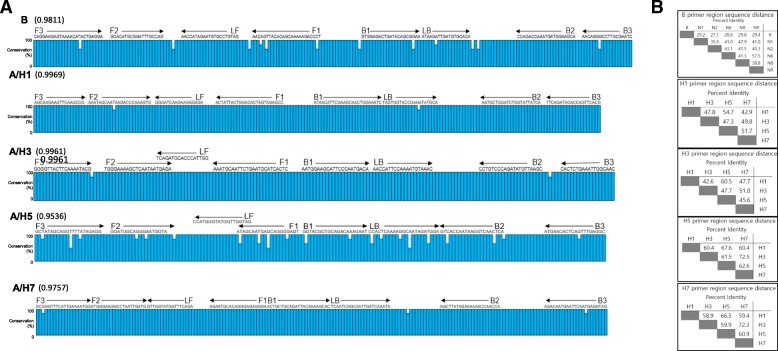
Highly conserved regions of HA (Influenza A H1, H3, H5, H7) and NA (Influenza B viruses) genes used to design RT-LAMP primers. Nucleotide sequences from conserved regions within HA gene of Influenza A viruses (H1N1, H3N2, H5N1, H5N6, H5N8 and H7N9) and NA gene of Influenza B viruses were obtained using CLC Main workbench 7 (version 7.6.4.). **a** Primer mapping (primer conversation average). These primers were designed to roughly 200 bp of this conserved sequence. **b** Sequence homology among target regions. The primer region sequence distance of Influenza virus B was calculated by comparing with NA subtype gene. H1, H3, H5, and H7 of Flu A were calculated for each primer region sequence distance of HA. For more information on Influenza virus RT-LAMP primer sequences, see Table 1

More than 200 HA gene sequences of H1N1, H3N2, H5N1, H5N6, H5N8, and H7N9 with NA gene sequences of influenza B viruses (both Victoria and Yamagata lineages) were downloaded from NCBI Influenza Virus Resource Database (https://www.ncbi.nlm.nih.gov/genomes/FLU) and analyzed with CLC Genomics Workbench to identify highly conserved regions within similar subtypes distinct from other subtypes (Fig. 1). RT-LAMP primer sets for each influenza subtype were designed using LAMP primer design software (Primer Explorer V4). Primer sets included two external primers (forward outer primer F3 and backward outer primer B3), two internal primers (forward inner primer FIP and backward inner primer BIP), and two loop primers (backward loop primer LB and forward loop primer LF) to augment the number of loops in the LAMP reaction, thus enhancing its reaction speed. Type B influenza-specific primers were designed to be specific for NA gene based on consensus sequence identified in 200 sequences of its two lineages (Victoria and Yamagata). Alternatively, influenza subtype-specific primers were designed to be specific to HA gene based on consensus sequences identified among 200 random sequences for each of H1N1, H3N2, H5N1, H5N6, H5N8, and H7N9 subtypes. All primers were synthesized by Cosmogenetech (Republic of Korea). Detailed information for all five primer sets used in this study is presented in Table 1.

**Table 1:** Reverse transcriptional loop-mediated isothermal amplification (RT-LAMP) primers for detection of influenza subtypes

Target	Gene	Primer name	Sequence (5′-3′)	Primer final concentration (μM)	Gene position	Length (mer)
B	NA	B-F3	CAGGAAGAGTAAAACATACTGAGGA	5	856–881	25
		B-B3	GATTCGCAAGGCCCTGTT	5	1052–1069	18
		B-FIP	AGGGTCTTTTTGCTGTGTAACTGTT-GCACATGCGGATTTGCCAG	40	933–955 + 883–901	44
		B-BIP	GTGGAGACTGATACAGCGGAA-TGCTTCCATCATTTGGTCTGG	40	972–992 + 1030–1050	42
		B-LF	GATGTCCGTGTAAGATACCAA	10	908–928	21
		B-LB	ATAAGATTGATGTGCACA	10	993–1010	18
A/H1	HA	H1-F3	AGCAAGAAGTTCAAGCCG	5	619–639	18
		H1-B3	CGTGAACTGGTGTATCTGAA	5	801–820	20
		H1-FIP	GGCTCTACTAGTGTCCAGTAATAGT-AAATAGCAATAAGACCCAAAGTG	80	734–758 + 689–711	48
		H1-BIP	ATAACATTCGAAGCAACTGGAAATC-TGATAATACCAGATCCAGCATT	80	718–742 + 778–799	47
		H1-LF	TCTCCCTTCTTGATCCC	10	713–729	17
		H1-LB	TAGTGGTACCGAGATATGCA	10	794–813	20
A/H3	HA	H3-F3	GGGGTTACTTCAAAATACG	5	841–859	19
		H3-B3	GTTGCCAATTTCAGAGTG	5	1011–1028	18
		H3-FIP	GAGTGATGCATTCAGAATTGCATTT-TGGGAAAAGCTCAATAATGAGA	40	903–927 + 863–884	47
		H3-BIP	AATGGAAGCATTCCCAATGACA-GCTTAACATATCTGGGACAGG	40	930–951 + 988–1008	43
		H3-LF	CCAATGGGTGCATCTGA	10	885–901	17
		H3-LB	AACCATTCCAAAATGTAAAC	10	952–971	20
A/H5	HA	H5-F3	GCTATAGCAGGTTTTATAGAGG	5	1048–1069	22
		H5-B3	GCCTCAAACTGAGTGTTCAT	5	1210–1229	20
		H5-FIP	ACTCCCCTGCTCATTGCTAT-GGATGGCAGGGAATGGTA	80	1112–1131 + 1072–1089	38
		H5-BIP	GGTACGCTGCAGACAAAGAAT-TGAGTTGACCTTATTGGTGAC	80	1133–1153 + 1177–1197	42
		H5-LF	CTACCAACCATACCCATGG	5	1093–1114	19
		H5-LB	CCACTCAAAAGGCAATAGATGGA	5	1154–1176	23
A/H7	HA	H7-F3	GCGGGTTTCATTGAAAATGG	5	1036–1055	20
		H7-B3	CTACCTCATTGAATTCATTGTCT	5	1215–1237	23
		H7-FIP	TCCCTCTCCCTGTGCATTCT-ATGGGAAGGCCTAATTGATG	80	1097–1116 + 1056–1075	40
		H7-BIP	ACTGCTGCAGATTACAAAAGCAC-TGGTTGGTTTTTTCTATAAGCC	80	1117–1139 + 1178–1199	45
		H7-LF	TCTGAAACCATACCAAC	5	1076–1092	17
		H7-LB	TCAATCGGCAATTGATCAAATA	5	1140–1161	22

### Optimization of RT-LAMP reaction conditions for each influenza subtype

To optimize the sensitivity and specificity of RT-LAMP detection, various primer concentrations (2.5 to 20 μM for external primer F3 and B3, 20 to 80 μM for internal primer FIP and BIP, and 5 to 20 μM for loop primer LF and LB) were used. For RT-LAMP reactions, a master mix solution was prepared containing 5 μl of WarmStart® Colorimetric LAMP 2X Master Mix (NEB, UK), 1 μl of each F3 and B3 primer, 1 μl of each FIP and BIP primer, and 1 μl of each LF and LB primer for each reaction. Optimized final concentration of each primer is presented in Table 1. Then 2 μl of RNA template extracted from virus was added to the master mix. The RT-LAMP reaction was performed at 65 °C for 60 min. It was then heated at 80 °C for 10 min for enzyme inactivation in a heat block. Positive RT-LAMP reactions resulted in a color change of phenol red pH indicator from pink to yellow due to decreased pH in the presence of extensive DNA polymerase activity. Thus, results could be directly observed by naked eyes. RT-LAMP results were also confirmed by 2% agarose gel electrophoresis.

### Comparing sensitivities of multiplex RT-LAMP, conventional one-step RT-PCR, and quantitative (q)RT-PCR

To compare sensitivity of our multiplex RT-LAMP assay to those of conventional one-step RT-PCR and qRT-PCR approaches, extracted RNA samples were serially diluted 10-fold to 10^− 6^. Then 2 μl of serially diluted RNA was mixed with the optimized master mix and subjected to RT-LAMP as described above. One-step RT-PCR was performed using 10 pmol of outer primers (F3 and B3) and equal amount of serially diluted RNA template as used for RT-LAMP with TOPscript™ One-step RT-PCR Kit (Enzynomics, Republic of Korea) under the following conditions: reverse transcription at 55 °C for 30 min, initial denaturation at 95 °C for 5 min, 35 cycles of denaturation at 95 °C for 30s, annealing at 60 °C for 30s, elongation at 72 °C for 30s, and a final elongation step at 72 °C for 5 min. qRT-PCR was performed using TOPreal™ One-step RTqPCR Kit (SYBR Green, low Rox) (Enzynomics, Republic of Korea) with 10 pmol of outer primers (F3 and B3) and equal RNA template as used for RT-LAMP. PCR conditions were the same as those performed for the one-step RT-PCR described above using a real-time machine, CFX96 TouchTM Real-Time PCR Detection System (Bio-Rad, Hercules, CA, USA). Results of RT-LAMP and one-step RT-PCR were visually confirmed using 2% agarose gel electrophoresis.

### Specificity of the multiplex RT-LAMP assay

To determine the specificity of the optimized multiplex RT-LAMP assay for targeting tested influenza viruses (type B, H1, H3, H5, and H7), RNA samples of other subtypes of influenza viruses including A/Em/Korea/W357/2008 (H2N3), A/Em/Korea/W210/2007 (H4N4), A/Em/Korea/W502/2015 (H6N2), A/Em/Korea/W563/2016 (H8N6), A/Em/Korea/W233/2007 (H9N2), A/Em/Korea/W372/2008 (H10N7), A/Em/Korea/W552/2016 (H11N9), and A/Em/Korea/W373/2008 (H12N5) were assessed using the multiplex RT-LAMP assay described in this study. In addition, one-step RT-PCR or qRT-PCR was performed to confirm the presence of viral genomic RNA using specific primer sets (Additional file 1: Table S1). One-step RT-PCR conditions were the same as described above. Results of one-step RT-PCR were confirmed using 2% agarose gel electrophoresis.

### Clinical evaluation

A total of 73 influenza-positive (confirmed using a qRT-PCR method) clinical nasopharyngeal aspirate samples collected from patients who demonstrated flu-like symptoms at Chungbuk National University Hospital, Republic of Korea were used for clinical evaluation of the RT-LAMP diagnostic assay developed in this study. In addition, 18 spiked samples in which 10^4^ TCID_50_/ml of viruses (i.e., H1N1, H5N6, H5N8, and H7N9) were diluted into flu-negative human nasopharyngeal aspirate samples were used for clinical evaluation. RNA was extracted from clinical/spiked samples and subjected to multiplex RT-LAMP and conventional RT-PCR for comparative detection of specific influenza viruses. To verify the specificity of the RT-LAMP assay using clinical samples, 44 RNA samples of human infectious viruses other than those targeting influenza viruses including human enterovirus (HEV), adenovirus (AdV), parainfluenza virus (PIV), human metapneumovirus (MPV), human bocavirus (HboV), human rhinovirus (HRV), human coronavirus 229E (229E), human coronavirus NL63 (NL63), human coronavirus OC43 (OC43), respiratory syncytial virus A (RSVA), respiratory syncytial virus B (RSVB), and Middle East Respiratory Syndrome coronavirus (MERS-CoV) were subjected to multiplex RT-LAMP assay. RNA samples of all other human infectious viruses except MERS-CoV were extracted from clinical swab or nasopharyngeal aspirate samples collected from patients and confirmed positive by qRT-PCR. RNA samples from MERS-CoV grown in cell culture were extracted and diluted in human nasopharyngeal aspirate as additional samples for spiked test.

## Results

### Optimization of RT-LAMP assay

**Fig. 2:**
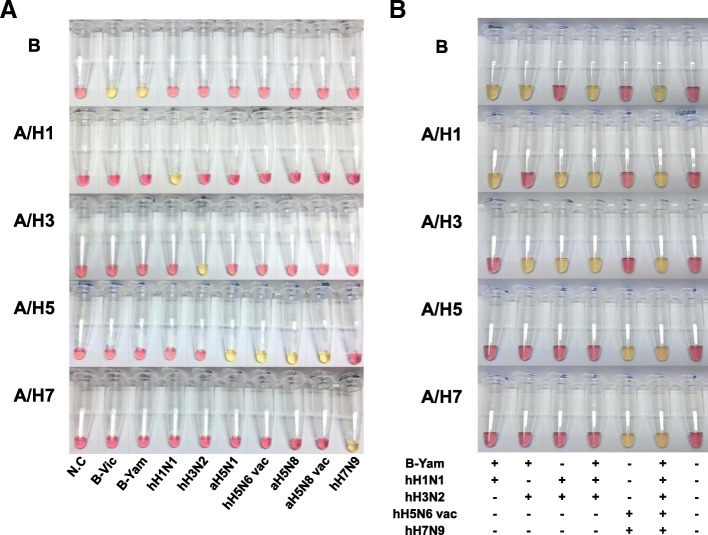
Specificity of influenza RT-LAMP. To evaluate specificity of each RT-LAMP primer set, RT-LAMP was performed to assess cross-reactivity using (**a**) individual and (**b**) mixed influenza virus samples. RT-LAMP reactions were conducted by incubation at 65 °C for 1 h. Positive RT-LAMP reactions resulted in a color change from pink to yellow. For confirmation of colorimetric RT-LAMP, see image of agarose gel electrophoresis in Additional file 1: Figure S1. B-Vic: B/Brisbane/60/2008 (Victoria lineage); B-Yam: B/Phuket/3073/2013 (Yamagata lineage); hH1N1: A/California/04/2009; H3N2: A/Perth/16/2009; aH5N1: A/Em/Korea/w149/2006; hH5N6 vac: A/Sichuan/26221/2014; aH5N8: A/Em/Korea/w468/2014; aH5N8 vac: A/gyrfalcon/Washington/41088–6/2014; hH7N9: A/Anhui/1/2013; N.C.: Negative control (D.W.)

Following standardization and optimization, final concentrations of each primer set were fixed to be 5 μM for F3 and B3, 40 μM for FIP and BIP, and 10 μM for LF and LB for detecting type B and H3 subtypes, 5 μM for F3 and B3, 80 μM for FIP and BIP, and 10 μM for LF and LB for detecting the H1 subtype, and 5 μM for F3 and B3, 80 μM for FIP and BIP, 5 μM for LF and LB for detecting H5 and H7 subtypes (Table 1). Targeted genes of specific subtypes were successfully amplified using these designed RT-LAMP primers and observed by color changes within RT-LAMP reaction tubes (Fig. 2a). A change in color from pink to light yellow indicated a positive reaction while negative reactions retained a pink color (Fig. 2). The specificity of these RT-LAMP primers was additionally verified using mixed virus samples in various combinations. Targeted genes in mixed virus samples were also specifically amplified by RT-LAMP primers (Fig. 2b). In addition, agarose gel electrophoresis of RT-LAMP products showed a typical ladder-like banding pattern (Additional file 1: Figure S1). These results indicate that multiplex RT-LAMP primers described here can efficiently and specifically detect corresponding subtypes. They can also detect multiple human influenza virus genes as a universal element in this RT-LAMP assay.

### Sensitivity of multiplex RT-LAMP assay for human influenza viruses and avian influenza viruses infecting humans compared to conventional PCR-based assays

To determine the detection limit of the multiplex RT-LAMP assay for human influenza viruses and avian influenza viruses infecting humans, we performed amplification of endpoint-diluted viral RNA extracted from each virus after propagation in 11-day-old chicken embryonic eggs for 48–72 h at 33 °C for type B viruses and 37 °C for type A viruses. RNA of each virus was serially diluted (10-fold up to 10^− 6^) in distilled water (DW). Infectious viral particles were determined using TCID_50_/ml. Infectious viral genome copies per microliter of extracted RNA were estimated and presented in Table 2. As shown in Table 2 and Additional file 1: Figure S2, the multiplex RT-LAMP assay revealed a detection limit range of 0.1 ~ 100 viral genome copies (*p* < 0.05) for all human influenza viruses and avian influenza viruses infecting humans in our panel [i.e., type B (Victoria- and Yamagata-lineages), seasonal H1N1, H3N2, highly pathogenic avian influenza H5N1, H5N6, and H5N8 isolated from human and avian species, and human H7N9 viruses]. On the other hand, the conventional one-step RT-PCR and real-time RT-PCR assays demonstrated detection limits ranging from 0.1 ~ 1,000 infectious viral genome copies for the same viral RNA samples (Table 2 and Additional file 1: Figure S2). Total reaction and observation time of conventional PCR-based detection methods took 2~3 h while colorimetric visualization of multiplex RT-LAMP assay results took only 1 h. Overall, these results demonstrate that the multiplex RT-LAMP assay developed in this study targeting HA or NA gene of influenza viruses exhibits similar or higher sensitivity with reduced detection time compared to conventional PCR-based assays.

**Table 2:**
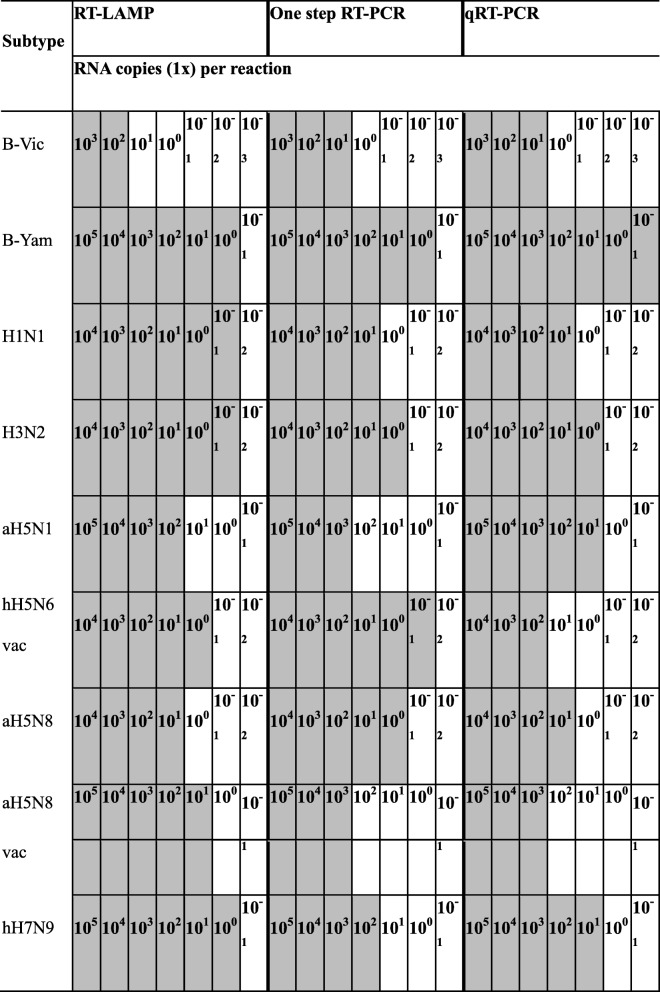
Sensitivity of the RT-LAMP assay compared with conventional methods

The gray-colored block represents the positive reactions of the assays at the dilutin point of RNA sample.

*B-Vic* B/Brisbane/60/2008 (Victoria lineage), *B-Yam* B/Phuket/3073/2013 (Yamagata lineage), *H1N1* A/California/04/2009, *H3N2* A/Perth/16/2009, *aH5N1* A/Em/Korea/w149/2006, *hH5N6 vac* A/Sichuan/26221/2014, *aH5N8* A/Em/Korea/w468/2014, *aH5N8 vac* A/gyrfalcon/Washington/41088–6/2014, *hH7N9* A/Anhui/1/2013

### Specificity of the multiplex RT-LAMP assay determined by assessing cross-reactivity to other influenza subtypes and human infectious viruses

**Fig. 3:**
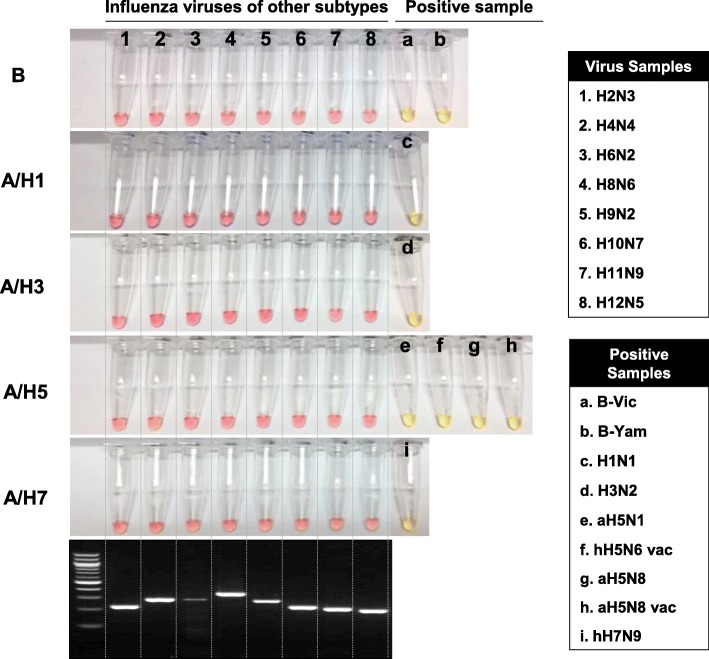
Specificity of Influenza RT-LAMP compared to other subtypes of influenza viruses. RT-LAMP reactions were performed using RNA from H2N3, H4N4, H6N2, H8N6, H9N2, H10N7, H11N9, and H12N5 and each primer set (B, A/H1, A/H3, A/H5, A/H7) to evaluate whether this RT-LAMP assay could cross-react with other influenza virus subtypes. RNAs from other influenza virus subtypes were confirmed using 1-step RT-PCR. Please see Additional file 1: Table S1 for additional details of one-step RT-PCR primer sequences

We also examined the specificity of the multiplex RT-LAMP assay using other subtypes of influenza (H2, H4, H6, H9, H10, H11, and H12) and other human infectious viruses obtained from a total of 44 clinical/spiked samples positive for HEV, AdV, PIV, MPV, HboV, HRV, 229E, NL63, OC43, RSVA, RSVB, and MERS-CoV that might potentially be in co-circulation with target viruses and cause similar symptoms. Based on colorimetric assessments, the RT-LAMP reaction using each primer set for H1, H3, H5, and H7, and type B viruses demonstrated no cross-reactivity with other influenza subtypes or human infectious viruses tested (Fig. 3 and Table 3). Conventional RT-PCR and qRT-PCR assays using specific primers (Additional file 1: Table S1) for other influenza subtypes and human infectious viruses were also performed to verify the presence of corresponding viral RNA. Target gene amplification was observed (Fig. 3 and Table 3). These results indicate that the RT-LAMP assay can specifically detect influenza viruses tested in the current study.

**Table 3:** Specificity of multiplex influenza RT-LAMP assay in other human infectious viruses

Virus	Number of samples	RT-LAMP					qRT-PCR (Ct value)
		B	H1	H3	H5	H7	
HEV	1	–	–	–	–	–	31.5
AdV	2	–	–	–	–	–	29.08–33.85
PIV	6	–	–	–	–	–	18.27–33.41
MPV	5	–	–	–	–	–	23.16–37.5
HboV	1	–	–	–	–	–	33.25
HRV	5	–	–	–	–	–	26.12–34.7
229E	5	–	–	–	–	–	19.55–33.48
NL63	3	–	–	–	–	–	19.71–23.6
OC43	5	–	–	–	–	–	21.24–35.57
RSVA	5	–	–	–	–	–	20.27–31.06
RSVB	5	–	–	–	–	–	17.18–27.78
MERS^a^	1	–	–	–	–	–	15.9

*HEV* human enterovirus, *AdV* adenovirus, *PIV* parainfluenza virus, *MPV* human metapneumovirus, *HboV* human bocavirus, *HRV* human rhinovirus, *229E* human coronavirus 229E, *NL63* human coronavirus NL63, *OC43* human coronavirus OC43, *RSVA* respiratory syncytial virus A, *RSVB* respiratory syncytial virus B, *MERS* Middle East respiratory syndrome coronavirus

^a^MERS: spiked sample; Ct: cycle threshold

### Evaluation of the multiplex RT-LAMP assay using clinical and spiked samples

A total of 73 clinical samples (H1N1, *n* = 3; H3N2, *n* = 35; and type B, *n* = 35) and 18 spiked (H1N1, *n* = 9; H5N6, *n* = 4; H5N8, *n* = 4; and H7N9, *n* = 1) samples initially diagnosed with influenza using qRT-PCR (Ct values: 17.1~34.99) were clinically evaluated using the multiplex RT-LAMP assay developed in this study. For comparison, conventional RT-PCR was performed in parallel. Results showed that 97~100% of seasonal influenza viruses were specifically detected by the multiplex RT-LAMP which exhibited similar sensitivity to qRT-PCR: 35 out of 35 samples in type B (100%), 12 out of 12 samples in H1N1 (100%), and 34 out of 35 samples in H3N2 (97%) without cross-reaction to each other (Table 4). In addition, 100% of H5N6, H5N8, and H7N9 samples were detectable using this developed RT-LAMP assay. However, 83~100% of influenza viruses in clinical and spiked samples were detected using conventional RT-PCR, showing greater that 6% reduction in sensitivity compared to the RT-LAMP assay (Table 4). These results suggest that the multiplex RT-LAMP diagnostic assay described in this study is as sensitive as qRT-PCR. However, it provides results more rapidly with greater simplicity.

**Table 4:** Performance of the multiplex RT-LAMP assay and one-step RT-PCR for influenza virus detection using clinical and spiked samples based on qRT-PCR assay

Subtype	sample type	qRT-PCR (Ct value)	Positive rate % (positive number/test number)	
			RT-LAMP	RT-PCR
B	clinical	17.1–34.99	100% (35/35)	94% (33/35)
H1N1	clinical/spiked^a^	19.61–32.6	100%(12/12)	83%(10/12)
H3N2	clinical	21.79–34.89	97%(34^b^/35)	91%(32/35)
H5N6/H5N8	spiked	22.2–24.54	100%(8/8)	100%(8/8)
H7N9	spiked	21.25	100%(1/1)	100%(1/1)
Total		< 35	98.9%(90/91)	92.3%(84/91)
Time required		120 min	60 min	170 min^c^

*Ct* cycle threshold

^a^H1N1: clinical sample (3), spiked sample (9)

^b^1 sample undetected by RT-LAMP was confirmed with H3N2 by sequencing

^c^Including gel electrophoresis time for confirmation of results

## Discussion

Influenza virus, an acute respiratory infectious agent, can rapidly propagate in the upper respiratory tract. It is capable of airborne transmission to other individuals [33]. Prescribing anti-influenza drugs following rapid and accurate diagnosis of an infection is thus critical to mitigate viral spread in the early stage of an outbreak. Many primary care providers use RIDT to diagnose influenza infections because it is simple to use with relatively rapid results. However, the accuracy of this approach is lower than that of qRT-PCR. A highly accurate gene-based diagnostic assay would serve as a valuable local on-site tool if it is easy to use with rapid results. Although many CLIA-waived molecular tests satisfy these criteria due to recent advancement, most of these developed tests are limited to detect seasonal influenza without appropriate subtype discrimination. In this study, we developed a multiplex RT-LAMP method to distinctively diagnose human influenza (e.g., seasonal influenza type B, H1N1, H3N2) and avian influenza viruses infecting humans (e.g., H5N1, H5N6, and H7N9). Hence, this developed method would be essential in some countries where these viruses are co-circulating in humans. Moreover, the RT-LAMP method described here can be used to intuitively detect these viruses using a one-pot colorimetric visualization approach, making it a more feasible POCT.

Our multiplex RT-LAMP detection system was designed to be a more feasible RIDT with accuracy as high as RT-PCR methods for detecting most recent human influenza viruses and avian influenza viruses infecting humans. Although a variety of reliable and affordable RT-LAMP methods have been developed to detect human or avian influenza viruses, each can only detect an individual subtype [23–29, 34, 35] or limited subtypes of human influenza viruses and/or avian influenza viruses infecting humans [18, 30, 36, 37]. In a pandemic, it is critical to rapidly differentiate patients infected by seasonal flu from those infected by an emerging strain. Thus, reliable and affordable multiplex detection tools should be capable of identifying broad-spectrum influenza viruses infecting humans. Moreover, RT-LAMP method for most recent emerging HPAI H5N6 and H5N8 viruses which have high potential to infect humans has rarely been developed or evaluated for its detection efficacy. In addition to broad-spectrum detection of human influenza viruses using our multiplex RT-LAMP assay, we optimized commercially available colorimetric RT-LAMP enzyme. Previous studies have also shown that the colorimetric visualization system using dyes (e.g., SYBR) is detectable by naked eyes [38]. However, these colorimetric methods require additional steps (e.g., adding dye to test for color changes after reaction or the use of a UV device for visualization [39]) that can decrease their utility in resource-limited primary care settings.

RT-LAMP assay is one of promising diagnostics tools that can be utilized to empower disease detection in developing countries as it does not require sophisticated, expensive equipment or trained personnel. The complex design of each specific primer sets made to amplify target sequence with high degree of sensitivity and specificity is one of major recognized constraints of this assay. Recognizing its advantages when detecting RNA genome of viral pathogens, RT-LAMP assay still requires improvement, particularly for the RNA extraction step, to be an efficient and suitable field-based diagnostic tool. Current approaches for RNA extraction of nasopharyngeal swabs or aspirate specimens collected from influenza-suspected patients usually require laboratory processing equipment (e.g., use of centrifuge system and others laboratory devices), technical support, and electricity. Thus, the use of chaotropic salt extraction which does not require centrifugation [40] would improve the feasibility of the developed multiplex RT-LAMP method as it utilizes a syringe with RNA-binding filter method [41]. Furthermore, it has been reported that the RT-LAMP reaction could be performed without RNA extraction step for some RNA viruses [42]. Although our multiplex RT-LAMP assay has not been optimized to directly detect influenza viral RNA without RNA sample preparation, it would significantly increase its feasibility as a diagnostic tool for POCT.

Although RT-LAMP assays designed to detect Victoria lineage Type-B viruses has 10 times less sensitivity compared to one-step RT-PCR and qRT-PCR approaches, RT-LAMP reactions for other subtypes exhibited similar or higher sensitivity without cross-reaction to various human infectious viruses and other subtypes of avian influenza viruses. Furthermore, our multiplex RT-LAMP assay was more sensitive than a conventional RT-PCR approach using clinical or spiked samples. Although the RT-LAMP detection method for H7N9 was optimized and evaluated, single spiked sample for clinical verification would be a limitation of the method. The specificity of multiplex RT-LAMP assay demonstrated in this current study might have limitations for use as a primary detection method based on local epidemiological setting. However, this assay’s rapid detection has a valuable contribution by providing diagnostics specifically in areas with prevalent avian influenza viruses infecting humans.

## Conclusions

Our multiplex RT-LAMP method not only can minimize the use of expensive lab instruments and devices, but also can detect broad-spectrum human influenza viruses and avian influenza viruses infecting humans as accurately and more rapidly than conventional RT-PCR-based detection methods suitable for use in on-site testing. This method will improve and aid in the diagnosis of influenza infections and potentially increase the speed of clinicians to provide appropriate treatment.

## Additional file

Additional file 1 : **Figure S1.** Specificity of RT-LAMP. The specificity of the RT-LAMP assay was tested using (A) individual and (B) mixed influenza virus samples listed below. RT-LAMP amplicon was confirmed using 2% agarose gel electrophoresis. The positive sample (yellow color) has a typical ladder-like pattern of RT-LAMP reaction. (a) B-Vic:B/Brisbane/60/2008 (Victoria lineage); (b) B-Yam: B/Phuket/3073/2013 (Yamagata lineage); (c) H1N1:A/California/04/2009; (d) H3N2: A/Perth/16/2009; (e) aH5N1:A/Em/Korea/w149/2006; (f) hH5N6 vac: A/Sichuan/26221/2014; (g) aH5N8:A/Em/Korea/w468/2014; (h) aH5N8 vac:A/gyrfalcon/Washington/41088–6/2014; (i) hH7N9:A/Anhui/1/2013; N.C.: Negative control (D.W.). **Figure S2.** Sensitivity of the RT-LAMP assay compared with conventional methods. To estimate the sensitivity of the RT-LAMP assay, RNA samples from each influenza viruses were 10-fold serially diluted and used as templates for the RT-LAMP assay (A), conventional RT-PCR (B), and real-time qRT-PCR (C). RT-LAMP results are visualized colorimetrically and by gel-electrophoresis. Results of conventional RT-PCR (B) and real-time qRT-PCR (C) are visualized using gel electrophoresis and cycle threshold (Ct) values, respectively. Please see Table 2 for the full name of virus used. N.C., negative control. **Table S1.** Primers for detection of influenza viruses of other subtypes and human 3 respiratory disease viruses. (PDF 565 kb)

## Data Availability

All data generated or analyzed during this study are included in this published article and its supplementary information files.

## Abbreviations

229E: Human coronavirus 229E
AdV: Adenovirus
AIVs: Avian influenza viruses
B3: Backward outer primer
BIP: Backward inner primer
DW: Distilled water
F3: Forward outer primer
FIP: Forward inner primer
HA: Hemagglutinin
HboV: Human bocavirus
HEV: Human enterovirus
HPAI: Highly pathogenic avian influenza
HRV: Human rhinovirus
LB: Backward loop primer
LF: Forward loop primer
LPAI: Low pathogenic avian influenza
MEM: Minimal essential medium
MERS-CoV: Middle East Respiratory Syndrome coronavirus
MPV: Human metapneumovirus
NA: Neuraminidase
NAIs: Neuraminidase inhibitors
NL63: Human coronavirus NL63
OC43: Human coronavirus OC43
PIV: Parainfluenza virus
POCT: Point-of-care testing
qRT-PCR: Quantitative reverse transcriptional-polymerase chain reaction
RIDT: Rapid influenza detection tests
RSVA: Respiratory syncytial virus A
RSVB: Respiratory syncytial virus B
RT-LAMP: Reverse Transcriptional Loop-mediated Isothermal Amplification
TCID_50_: 50% tissue culture infectious dose
TPCK: L-(tosylamido-2-phenyl) ethyl chloromethyl ketone
