# Correction to: Rapid and simple colorimetric detection of multiple influenza viruses infecting humans using a reverse transcriptional loopmediated isothermal amplification (RT-LAMP) diagnostic platform

**DOI:** 10.1186/s12879-020-05707-y

**Published:** 2020-12-24

**Authors:** Su Jeong Ahn, Yun Hee Baek, Khristine Kaith S. Lloren, Won-Suk Choi, Ju Hwan Jeong, Khristine Joy C. Antigua, Hyeok-il Kwon, Su-Jin Park, Eun-Ha Kim, Young-il Kim, Young-Jae Si, Seung Bok Hong, Kyeong Seob Shin, Sungkun Chun, Young Ki Choi, Min-Suk Song

**Affiliations:** 1 Department of Microbiology, College of Medicine and Medical Research, Institute, Chungbuk National University, Chungdae-ro 1, Seowon-Ku, Cheongju, 28644 Republic of Korea; 2 Department of Clinical Laboratory, Science, Chungbuk Health and Science University, Cheongju, Republic of Korea; 3 Departments of Laboratory Medicine, Chungbuk National University, College of Medicine, Cheongju, Republic of Korea; 4 Department of Physiology, Chonbuk National University Medical School, Jeonju, 54907 Republic of Korea

**Correction to: BMC Infect Dis 19, 676 (2019)**

**https://doi.org/10.1186/s12879-019-4277-8**

Following publication of the original article [1], the authors noticed that in Table 1, two primer sequences (B-LF and H5-LF) should have been reverse complemented.

**Table 1:** Reverse transcriptional loop-mediated isothermal amplification (RT-LAMP) primers for detection of influenza subtypes

Target	Gene	Primer name	Sequence(5′-3′)	Primer final concentration	Gene position	Length
						(mer)
B	NA	B-F3	CAGGAAGAGTAAAACATACTGAGGA	5 μM	856–881	25
		B-B3	GATTCGCAAGGCCCTGTT	5 μM	1052–1069	18
		B-FIP	AGGGTCTTTTTGCTGTGTAACTGTT-GCACATGCGGATTTGCCAG	40 μM	933–955 + 883–901	44
		B-BIP	GTGGAGACTGATACAGCGGAA-TGCTTCCATCATTTGGTCTGG	40 μM	972–992 + 1030–1050	42
		B-LF	CTACAGGCACATTCTATGGTT	10 μM	908–928	21
		B-LB	ATAAGATTGATGTGCACA	10 μM	993–1010	18
A/H1	HA	H1-F3	AGCAAGAAGTTCAAGCCG	5 μM	619–639	18
		H1-B3	CGTGAACTGGTGTATCTGAA	5 μM	801–820	20
		H1-FIP	GGCTCTACTAGTGTCCAGTAATAGT-AAATAGCAATAAGACCCAAAGTG	80 μM	734–758 + 689–711	48
		H1-BIP	ATAACATTCGAAGCAACTGGAAATC-TGATAATACCAGATCCAGCATT	80 μM	718–742 + 778–799	47
		H1-LF	TCTCCCTTCTTGATCCC	10 μM	713–729	17
		H1-LB	TAGTGGTACCGAGATATGCA	10 μM	794–813	20
A/H3	HA	H3-F3	GGGGTTACTTCAAAATACAAG	5 μM	841–859	19
		H3-B3	GTTGCCAATTTCAGAGTGTT	5 μM	1011–1028	18
		H3-FIP	GAGTGATGCATTCAGAATTGCATTT-TGGGAAAAGCTCAATAATGAGA	40 μM	903–927 + 863–884	47
		H3-BIP	AATGGAAGCATTCCCAATGACA-GCTTAACATATCTGGGACAGG	40 μM	930–951 + 988–1008	43
		H3-LF	CCAATGGGTGCATCTGA	10 μM	885–901	17
		H3-LB	AACCATTCCAAAATGTAAAC	10 μM	952–971	20
A/H5	HA	H5-F3	GCTATAGCAGGTTTTATAGAGG	5 μM	1048–1069	22
		H5-B3	GCCTCAAACTGAGTGTTCAT	5 μM	1210–1229	20
		H5-FIP	ACTCCCCTGCTCATTGCTAT-GGATGGCAGGGAATGGTA	80 μM	1112–1131 + 1072–1089	38
		H5-BIP	GGTACGCTGCAGACAAAGAAT-TGAGTTGACCTTATTGGTGAC	80 μM	1133–1153 + 1177–1197	42
		H5-LF	GGTACCCATACCAACCATC	5 μM	1093–1114	19
		H5-LB	CCACTCAAAAGGCAATAGATGGA	5 μM	1154–1176	23
A/H7	HA	H7-F3	GCGGGTTTCATTGAAAATGG	5 μM	1036–1055	20
		H7-B3	CTACCTCATTGAATTCATTGTCT	5 μM	1215–1237	23
		H7-FIP	TCCCTCTCCCTGTGCATTCT-ATGGGAAGGCCTAATTGATG	80 μM	1097–1116 + 1056–1075	40
		H7-BIP	ACTGCTGCAGATTACAAAAGCAC-TGGTTGGTTTTTTCTATAAGCC	80 μM	1117–1139 + 1178–1199	45
		H7-LF	TCTGAAACCATACCAAC	5 μM	1076–1092	17
		H7-LB	TCAATCGGCAATTGATCAAATA	5 μM	1140–1161	22

The sequences to correct are below marked:

The correct sequences should be indicated as below:

**Table:** 

**No.**	**Needs Correction:**		**Corrected version**
1.	Table 1/ B (NA gene)/B-LF Sequence:	GATGTCCGTGTAAGATACCAA	CTACAGGCACATTCTATGGTT
**Comment:** Based on the reference in designing the forward loop (LF) primer: **LF 5′-AACCATAGAATGTGCCTGTAG-3′** (Figure 1, page 4), this identified sequence should have been reverse complemented however inadvertently the submitted sequence for the mentioned B-LF primer was only reversed.			
2.	Table 1/ A/H5 (HA gene)/ H5-LF Sequence:	CTACCAACCATACCCATGG	GGTACCCATACCAACCATC
**Comment:** Based on the reference in designing the forward loop (LF) primer: **LF 5′–GATGGTTGGTATGGGTACC-3′** (Figure 1, page 4), this identified sequence should have been reverse complemented however inadvertently the submitted sequence for the mentioned H5-LF primer was only reversed.			
