# Supplementary material for: Rapid and simple colorimetric detection of multiple influenza viruses infecting humans using a reverse transcriptional loop-mediated isothermal amplification (RT-LAMP) diagnostic platform
Source: BMC Infect Dis. 2019 Aug 1;19:676. doi: 10.1186/s12879-019-4277-8 (PMC6669974; doi:10.1186/s12879-019-4277-8)
Supplement: Supplementary file 1 — : Figure S1. Specificity of RT-LAMP. The specificity of the RT-LAMP assay was tested using (A) individual and (B) mixed influenza virus samples listed below. RT-LAMP amplicon was confirmed using 2% agarose gel electrophoresis. The positive sample (yellow color) has a typical ladder-like pattern of RT-LAMP reaction. (a) B-Vic:B/Brisbane/60/2008 (Victoria lineage); (b) B-Yam: B/Phuket/3073/2013 (Yamagata lineage); (c) H1N1:A/California/04/2009; (d) H3N2: A/Perth/16/2009; (e) aH5N1:A/Em/Korea/w149/2006; (f) hH5N6 vac: A/Sichuan/26221/2014; (g) aH5N8:A/Em/Korea/w468/2014; (h) aH5N8 vac:A/gyrfalcon/Washington/41088–6/2014; (i) hH7N9:A/Anhui/1/2013; N.C.: Negative control (D.W.). Figure S2. Sensitivity of the RT-LAMP assay compared with conventional methods. To estimate the sensitivity of the RT-LAMP assay, RNA samples from each influenza viruses were 10-fold serially diluted and used as templates for the RT-LAMP assay (A), conventional RT-PCR (B), and real-time qRT-PCR (C). RT-LAMP results are visualized colorimetrically and by gel-electrophoresis. Results of conventional RT-PCR (B) and real-time qRT-PCR (C) are visualized using gel electrophoresis and cycle threshold (Ct) values, respectively. Please see Table 2 for the full name of virus used. N.C., negative control. Table S1. Primers for detection of influenza viruses of other subtypes and human 3 respiratory disease viruses. (PDF 565 kb) [file 12879_2019_4277_MOESM1_ESM.pdf]

Supplementary Materials for:

Rapid and simple colorimetric detection of multiple influenza viruses infecting humans using a Reverse Transcriptional Loop-mediated Isothermal Amplification (RT-LAMP) diagnostic platform

Su Jeong Ahn, Yun Hee Baek, Khristine Kaith S. Lloren, Won-Suk Choi, Ju Hwan Jeong, Khristine Joy C. Antigua, Hyeok-il Kwon, Su-Jin Park, Eun-Ha Kim, Young-il Kim, Young-Jae Si, Seung Bok Hong, Kyeong Seob Shin, Sungkun Chun, Young Ki Choi, Min-Suk Song

Correspondence to: songminsuk@chungbuk.ac.kr

choiki55@chungbuk.ac.kr

Supplementary Figures

Figure S1

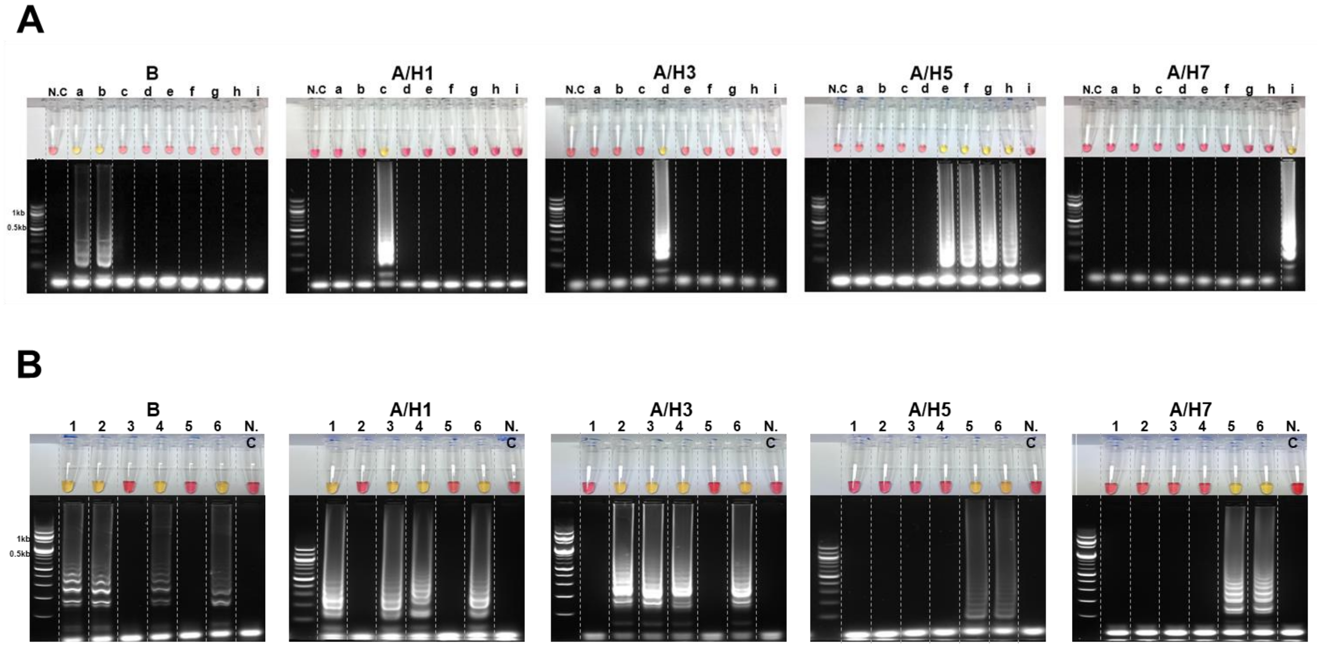

Virus	Co-infection spiked samples						
	1	2	3	4	5	6	N.C
B-Yam	+	+	-	+	-	+	-
hH1N1	+	-	+	+	-	+	-
hH3N2	-	+	+	+	-	+	-
hH5N6 vac	-	-	-	-	+	+	-
hH7N9	-	-	-	-	+	+	-

Figure S1. Specificity of RT-LAMP. The specificity of the RT-LAMP assay was tested using (A) individual and (B) mixed influenza virus samples listed below. The RT-LAMP amplicon was confirmed using 2% agarose gel electrophoresis. The positive sample (yellow color) has a typical ladder-like pattern of RT-LAMP reaction. (a) B-Vic:B/Brisbane/60/2008 (Victoria lineage); (b) B-Yam: B/Phuket/3073/2013 (Yamagata lineage); (c) H1N1:A/California/04/2009; (d) H3N2: A/Perth/16/2009; (e) aH5N1:A/Em/Korea/w149/2006; (f) hH5N6 vac: A/Sichuan/26221/2014; (g) aH5N8:A/Em/Korea/w468/2014; (h) aH5N8 vac:A/gyrfalcon/Washington/41088-6/2014; (i) hH7N9:A/Anhui/1/2013; N.C: Negative control (D.W)

Figure S2

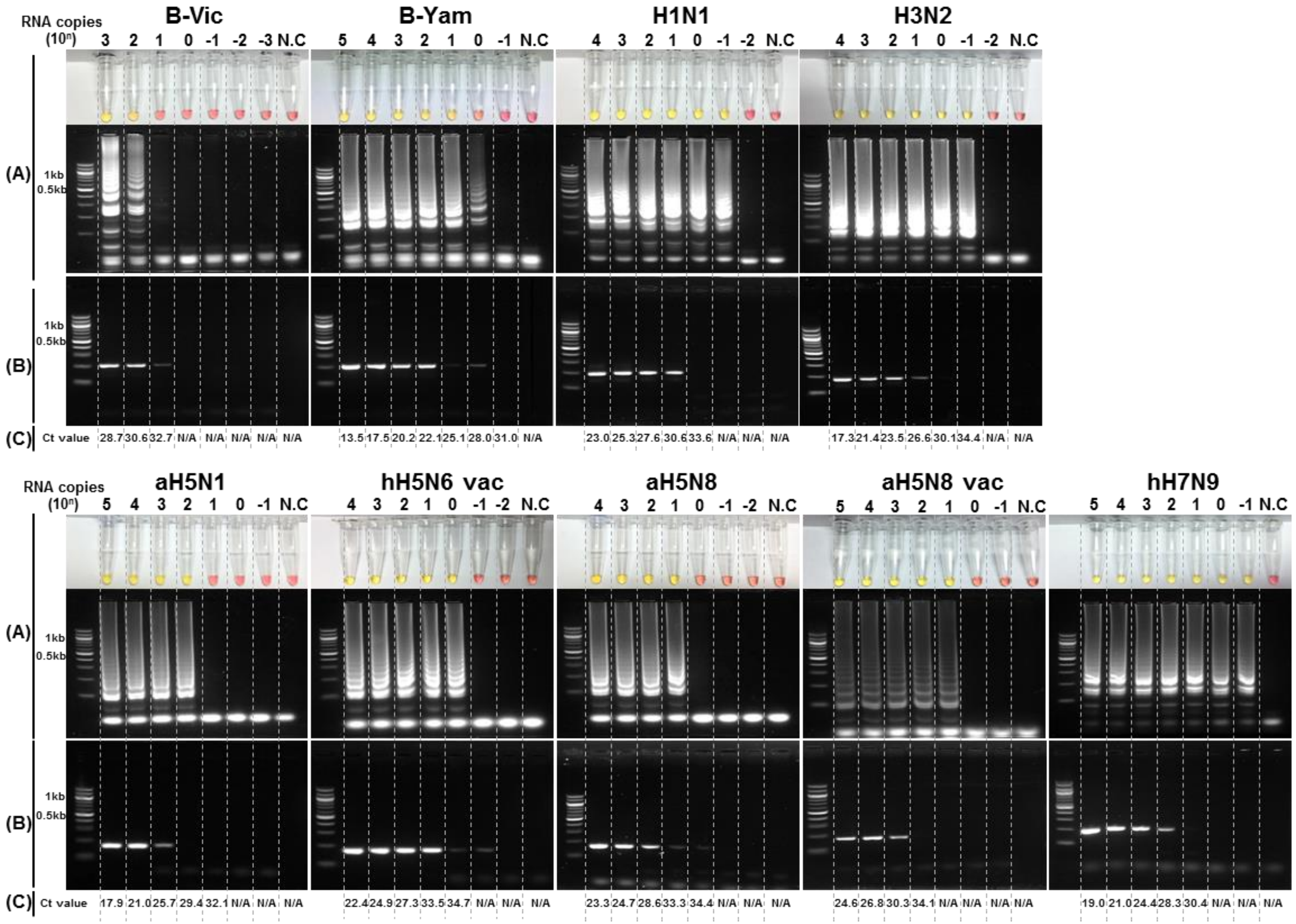
RNA copies (10^n)
B-Vic
3 2 1 0 -1 -2 -3 N.C
B-Yam
5 4 3 2 1 0 -1 N.C
H1N1
4 3 2 1 0 -1 -2 N.C
H3N2
4 3 2 1 0 -1 -2 N.C
(A)
1kb
0.5kb
(B)
1kb
0.5kb
(C) Ct value
28.7 30.6 32.7 N/A N/A N/A N/A N/A
13.5 17.5 20.2 22.1 25.1 28.0 31.0 N/A
23.0 25.3 27.6 30.6 33.6 N/A N/A N/A
17.3 21.4 23.5 26.6 30.1 34.4 N/A N/A
RNA copies (10^n)
aH5N1
5 4 3 2 1 0 -1 N.C
hH5N6 vac
4 3 2 1 0 -1 -2 N.C
aH5N8
4 3 2 1 0 -1 -2 N.C
aH5N8 vac
5 4 3 2 1 0 -1 N.C
hH7N9
5 4 3 2 1 0 -1 N.C
(A)
1kb
0.5kb
(B)
1kb
0.5kb
(C) Ct value
17.9 21.0 25.7 29.4 32.1 N/A N/A N/A
22.4 24.9 27.3 33.5 34.7 N/A N/A N/A
23.3 24.7 28.6 33.3 34.4 N/A N/A N/A
24.6 26.8 30.3 34.1 N/A N/A N/A N/A
19.0 21.0 24.4 28.3 30.4 N/A N/A N/A

Figure S2. Sensitivity of the RT-LAMP assay compared with conventional methods. To estimate the sensitivity of the RT-LAMP assay, RNA samples from each influenza virus were 10-fold serially diluted and used as templates for the RT-LAMP assay described here (A), conventional RT-PCR (B) and real-time qRT-PCR (C). RT-LAMP results are visualized colorimetrically and using gel-electrophoresis. The results of conventional RT-PCR (B) and real-time qRT-PCR (C) are visualized using gel electrophoresis and cycle threshold (Ct) values, respectively. Please see Table 2 for the full name of viruses used. N.C, negative control

Supplementary Tables

Table S1. Primers for detection of influenza viruses of other subtypes and human respiratory disease viruses.

Virus	Target	Primer name	Sequence(5'-3')
**Influenza viruses of other subtypes**	H2	H2-HA-199F	TTGGGAGATTGCAGCATCGC
		H2-HA-426R	TGTTGTATGCTGTGTCCACC
	H4	H4-HA-746F	TCGTAGAGCCTGGAGACCTA
		H4-HA-1057R	AAAGAGCCCTCTTGATGCCT
	H6	H6-HA-693F	CAGCAAGACCTGCTGTGAAC
		H6-HA-973R	CACATATTTGGGGCATTCTC
	H8	H8-HA-980F	AAAGCCTACGGCTGGCAGTT
		H8-HA-1316R	GAGGACCAGAAGCTCTGCAT
	H9	H9-HA-1262F	AGATTGATGACCAAATACAG
		H9-HA-1541R	ACCCCTTCTATTTTCTGCCT
	H10	H10-HA-256F	CATCTTACAGGGACATGGGA
		H10-HA-485R	AGCCACTTGAGCTCTGCGTA
	H11	H11-HA-503F	ACCAATCAGGAACATACCCT
		H11-HA-718R	CAGCCTGTCCATTAACTTTG
	H12	H12-HA-565F	GTATTCACATGGGCTATTCA
		H12-HA-753R	GTATCTTCACTGTCTGCCCT
**Human infectious disease viruses**	MERS-CoV	MERS F3-2	GGAATGGAATTAAGCAACTGGC
		MERS B3-2	CGCGAATTGTGTAACAATAGCT

MERS-CoV; Middle East Respiratory Syndrome Coronavirus (1)

Reference List

1. Lee SH, Baek YH, Kim Y-H, Choi Y-K, Song M-S, Ahn J-Y. 2017. One-pot reverse transcriptional loop-mediated isothermal amplification (RT-LAMP) for detecting MERS-CoV. Frontiers in microbiology 7:2166.
